# Dr. Close’s Paper: What Is the Vital Force?

**Published:** 1899-02

**Authors:** 


### DR. CLOSE'S PAPER: WHAT IS THE VITAL FORCE?

### DISCUSSION.

Dr. Patch—It is a great pity that Hahnemann did not, in his *Organon* give us clearer ideas as to just what ground he intended to cover by the phrase, "vital force." It is liable to misinterpretation. I do not understand from the phrases quoted from *The Organon*, that he meant to convey any disrespect to the vital force, but that he simply meant to define its limitations, and thereby to show the field for homœopathic remedies. The vital force, I think, is not supposed to be an intelligent force. There is a subtle difference between vital force as expressed by Hahnemann and Hudson's subjective mind. The exercise of choice, for instance, is not incumbent on the vital force, but on other functions. Dr.

Close, in his paper, mentioned the relation between all these lines of thought, but he did not bring out Swedenborg's particular ideas. I would like, with his permission, to show the wonderful correspondence between Hahnemann's vital force, as one may interpret it, and the animal spirit of Swedenborg. Swedenborg says, "The soul, apart from the *animal spirit*, could never have constructed the simple and middle organic forms of the body.

"The soul apart from the animal spirit could never produce the heart, or the vessels, either arterial or venous; or the red blood; or consequently the ultimate organic form of the body.

"Without the animal spirit the soul could determine nothing into action, and could do nothing in the body.

"Without the animal spirit the soul could feel none of the changes that happen to the body.

"The nature of the action and sensation, and even of the imagination and thought in an individual, are correspondent to the nature of the animal spirit and the circulation thereof in the body.

"The animal spirit makes us both spiritual and corporeal.

"In the human microcosm all that is above the animal spirit constitutes the inner man, and all that is below it, the outer.

"The animal spirit is never absolutely similar in any two individuals; on the contrary it is different in all the subjects of human society, and always different at different times in one and the same person.

"The animal spirit is that most pure humor which flows through the medullary fibres of the brain and the nervous fibres of the body.

"The animal spirit is the intermediate essence between the soul and the body, hence it is the mediatorial substance which provides for the communication of operations between the two.

"The animal spirit partakes of the essence of the soul, and of the essence of the body; that is to say, it is both spiritual and material.

"As the animal spirit is conceived and prepared in the cortical glands, it follows that the spiritual and material principles meet in it. The simple fibre arriving from its own simple cortex, pours into the minute chamber or cavity of the gland, a substance of the purest kind, which is conceived and born in the simple cortex—*i. e.*, the substance of the soul. And the finest vessels, which constitute the other portion of this simple or vascula medulla, supply a lymph or serum of the purest nature, capable of containing the purest corpuscles. From the marriage of these two substances the animal spirit is born."

Note the striking resemblance between Swedenborg's words and Hahnemann's, where, in sections nine to sixteen of *The Organon*, he describes the nature of the vital force. In his *Review of Physics*, also, there are several thoughts which are right along this line, and which further elucidate his meaning. These citations from Hahnemann illustrate the same thought. The Divine influx from on high, the impact of which is carried throught the efforts of the vital force to the corporeal body below, where it works out its ultimate purpose on earth.

The book by Hudson, quoted by Dr. Close, should be supplemented by a later work by the same author, *The Scientific Demonstration of a Future Life.* I think he shows there that the apparently blind action of the subjective consciousness becomes, under new conditions, and in a new atmosphere, the most potent part of the individual. While here it is under the control of the objective mind by which it is kept in subjection. In the future, after death, when the subjective mind becomes supreme and is rid of its material envelope, it unfolds into the immaterial, everliving soul.

Dr. Kennedy—Mr. Chairman, I feel somewhat the same as I did after Dr. Pease had finished. These are beautiful ideas that have been advanced; beautiful because they suggest not only what is possible, but probable, in the way of learning more of our being in the future. This one thought seems to me among the most beautiful: "Man is but a branch of the vine in the cosmic organism." In regard to the use of the words "force" and "power." Can we use those words interchangeably? Is not force power in action? I would like to ask, if, in this experiment of Dr. Gates, there was discovered a development of the individual brain cells, or a development by the multiplication of cells.

Dr. Close—Both. He stated that there was not only an increase in the number but in the size and complexity of the cells.

Dr. Kennedy—With regard to Hahnemann's higher human mind I get the idea that the higher human mind is rational. Is not the human mind rational in contradistinction from the mind of the lower animal?

Dr. Close—The word rational expresses the ability to reason, the ability to use the faculties intelligently. An animal reasons on a lower plane. Driving down DeKalb Avenue not long ago, behind a trolley car, my horse was brought to a stop by the stopping of the car. We frequently follow a car in the track. The horse stopped at a distance of about five feet from the rear end of the car, and stood watching the conductor and the people getting on and off. He is very observing, and much interested in the doings of his human friends. Several people were getting off. Before the last one or two passengers had gotten off the conductor reached his hand up to the bell rope, and the instant he did so my horse, who was watching him, started forward. Now why did he do that? The car had not yet started, but my horse had previously observed that it was the action of the conductor in pulling the bell rope which made the car start. He had reasoned that out. I call that a form of reasoning.

Dr. Pease—He certainly used reasoning powers there to the extent of judgment and will.

Dr. Kennedy—It brings up the old question of the difference between instinct and reason. We regard man as a being capable of judging between good and evil. We accept that as the standard of what is rational and human. Certainly it is the realm into which the lower animal does not enter. This idea of the development of the brain brought to my mind an incident related years ago by a professor of physiology, at that time at Boston University, who was very peculiar, to say the least, in some of his ideas. He claimed that he could, by examining the skull, tell the general characteristics of the man to whom the skull belonged. He claimed that the brain in activity developed more or less at the expense of the bony structure within, so that it was relatively thinner.

We are under the action of certain beneficent laws that so operate as to keep us in health. We may violate these laws at will, but the law still works. We have perverted its action. The power is there, but working in a wrong direction. We cannot blame the power. Through our wrongdoing, or the wrongdoing of some one living before us, the train is set going on the wrong track.

Dr. Pease—As I listened to the paper I could not but realize that there was not such a great fundamental difference between my own thought and that of Dr. Close, as I had begun to think. But I do differ from the ideas quoted from both Prof. Gates and Dr. Hudson and others. I, perhaps, may seem to show egotism, but it is not that. I predict that Prof. Gates and Dr. Hudson and many others of the mental philosophers and investigators of the present time will change their conclusions and deductions in the near future. Hahnemann, in common with other mental philosophers, makes the mistake of calling the manifestations of force the force itself. They use the word "mind" when they mean the soul or essence. They use the word spirit, soul, and mind interchangeably. They will learn the difference between the soul, mind, and spirit in the near future. They are coming to it just as surely as they prosecute their investigations.

In regard to Hahnemann's "blind, unreasoning force acting automatically," that assertion is inconsistent with the operations of our existence. I will concede the blind action in disease, but in health the vital force or power of life acts in accord with the laws of highest intelligence. Because we are not intelligently conscious of the power of life is no sign of lack of intelligent direction of its operations. The essence, the soul, furnishes the intelligence which directs the operation of this vital force. If the instrument, the body, becomes in any way imperfect in any of its functions or organs, then will that power of life be placed in a difficult position. It cannot operate intelligently through the imperfect instrument, or part of the instrument. The essence or substance must be adjusted harmoniously with the instrument which brings that essence to our physical sense. Dr. Close in his paper, and Prof. Gates and Dr. Hudson give illustrations of their observations of the vital operations, and of the director of these operations, the power of life. I tried to demonstrate, in my paper, the source of that control. Prof. Gates's experiments on animals kept under certain conditions prove the position I have taken in regard to the formative and causative influences of mind or of the emotions. By experimenting on these animals he developed the particular faculties under training and observation, and the faculties, through the power of life, caused the elaboration of brain tissue. With the deprivation of light in one experiment, the power of life could not act in that direction, and because there was no action, there was of necessity, no physical tissue expression of that action. All these results and operations observed by experimenters, I hold, are simply manifestations of the faculties of the soul, which, in their sum-total, constitute intelligence. The subjective and objective mind spoken of are mere phases of operation of the faculties. The subject under hypnotic suggestion simply realizes, for the time being, his conscious control of the power of life, and loans it, so to speak, to the will of the operator, and the power of life operates the subject and his faculties. Whether he is conscious or unconscious is another question. In the restoration of memory and its operation in fever, as in the case of the girl who unconsciously recited from memory what she had heard her master read at some past time, only the faculty of memory, and the faculties immediately related to it operated by reason of the fevered condition of the body. Reason cannot operate without the other faculties of the soul, either subjectively or objectively, by induction or deduction. Every normal form which has intelligence has faculties, and faculties can only act upon the physical structure of that form through the automatic, vital force. That force, I hold, must always be governed by the individuality or essence. In every animal form we meet with the different limitations to which they are subject. Those limitations differ according to the special use to be performed by intelligence, and are represented by the form in which that intelligence dwells.

Dr. Morgan—I don't know that I am able to add anything more than to ask a question about life. In the period of life from conception to birth, there seems to be going on a process of the finest architectural work of growth or structure formation, an organization of matter by some force. That force must come from or be governed by some intelligence, though the subject of the growth of the matter is entirely unconscious of it. The question with me is, What is it? I would like to hear some one give an explanation of that, whether it comes from mere chemical processes or from Divine intelligence. I do not profess to be able to solve the question.

Dr. Patch—Chemical processes would hardly account for the Divine creative instinct back of the pre-natal growth.

Dr. Kennedy—I suppose all these question are to be resolved back to the primary intelligence.

Dr. Morgan—This paper, as well as the discussion, has been of most intense interest to me. I would rather have quite a while to study it, however, before expressing any ideas.

Dr. Davis—I greatly enjoyed the paper, but will not take up time in discussing more than to ask a question. I thought the writer was about to carry the discussion into the vegetable kingdom. Is not the cellular action in the vegetable, in its process of development, governed by the same laws that govern the growth and development in the animal? Plants growing only a few feet from each other develop to be entirely different in color and shape and medical properties. Thus we get our different drugs, showing different properties when used. Can an action be called intelligent when it is not conscious?

Speaking of the development of tissue through the conscious action of the mind upon the brain cells recalls an instance which came under my notice where the nerve filaments of the finger had been examined, and there was found actual brain substance in these nerve filaments. It is abnormal, but it corresponded exactly to the development of the convolutions of the brain, as the writer stated. If the soul directs vital force, then all animals possess souls because all animals possess vital force.

Dr. Patch—Dr. Davis speaks of finding brain substance in the tactile corpuscles. Is it not a fact that all glands are analogous to the brain? All glands are similar in structure. Do they not really possess the same nature as brain substance?

Dr. W. S. Talcott—I think the verdict must be left with our psychologists. I do not know that they have taken it up, but as they pursue the subject in every way to determine the nature of the vital force and its action, I trust they will yet explain how results are accomplished. My own belief is that the vital force is a general one, controlling these mental manifestations which are peculiar to each individual. Statements should be made with care, while we look to our laboratories for a demonstration. We want the demonstration that practical experiment gives.

